# Sialyl Lewis x expression in canine malignant mammary tumours: correlation with clinicopathological features and E-Cadherin expression

**DOI:** 10.1186/1471-2407-7-124

**Published:** 2007-07-06

**Authors:** Salomé S Pinho, Augusto JF Matos, Célia Lopes, Nuno T Marcos, Júlio Carvalheira, Celso A Reis, Fátima Gärtner

**Affiliations:** 1 Institute of Molecular Pathology and Immunology of the University of Porto (IPATIMUP), Rua Dr Roberto Frias s/n, 4200-465 Porto, Portugal; 2 Institute of Biomedical Sciences of Abel Salazar (ICBAS), University of Porto, Largo Prof. Abel Salazar, 2, 4099-003 Porto, Portugal; 3 Medical Faculty, University of Porto, Alameda Prof. Hernâni Monteiro 4200-319 Porto, Portugal

## Abstract

**Background:**

Sialyl Lewis x (sLe^x^) antigen is a carbohydrate antigen that is considered not only a marker for cancer but also implicated functionally in the malignant behaviour of cancer cells. Overexpression of sLe^x ^is associated with enhanced progression and metastases of many types of cancer including those of the mammary gland. Canine mammary tumours can invade and give rise to metastases via either lymphatic or blood vessels.

E-Cadherin is specifically involved in epithelial cell-to-cell adhesion. In cancer, E-Cadherin underexpression is one of the alterations that characterizes the invasive phenotype and is considered an invasion/tumour suppressor gene. Partial or complete loss of E-Cadherin expression correlates with poor prognosis in canine malignant mammary cancer.

The aim of this study was to analyse the sLe^x ^expression in canine malignant mammary tumours and to evaluate if the presence of sLe^x ^correlates with the expression of E-Cadherin and with clinicopathological features.

**Methods:**

Fifty-three cases of canine mammary carcinomas were analysed immunohistochemically using monoclonal antibodies against sLe^x ^(IgM) and E-Cadherin (IgG). The clinicopathological data were then assessed to determine whether there was a correlation with sLe^x ^tumour expression. Double labelled immunofluorescence staining was performed to analyse the combined expression of sLe^x ^and E-Cadherin.

**Results:**

sLe^x ^expression was consistently demonstrated in all cases of canine mammary carcinomas with different levels of expression. We found a significant relationship between the levels of sLe^x ^expression and the presence of lymph node metastases. We also demonstrated that when E-Cadherin expression was increased sLe^x ^was reduced and vice-versa. The combined analysis of both adhesion molecules revealed an inverse relationship.

**Conclusion:**

In the present study we demonstrate the importance of sLe^x ^in the malignant phenotype of canine malignant mammary tumours. Our results support the use of sLe^x ^as a prognostic tumour marker in canine mammary carcinomas. Furthermore, we showed that sLe^x ^and E-Cadherin expression were inversely correlated. Future studies are warranted to clarify the molecular mechanism underlying the relation between sLe^x ^and E-Cadherin in canine mammary carcinoma cells which represents an important comparative model to woman breast cancer.

## Background

Mammary tumours are the most common tumours in intact female dogs and approximately 40% to 50% of these tumours are malignant [1]. All malignant canine mammary tumours have the potential to metastasise. In general canine malignant tumours metastasise via the lymphatics to the regional lymph nodes or hematogenously to the lungs that represent the most common site of distant metastases. [1-3]

Malignant transformation is associated with abnormal glycosylation, resulting in expression of altered carbohydrate determinants, such as the Sialyl Lewis x (sLe^x^) antigen. Altered cell surface glycosylation is a prominent feature of malignant tumour cells and define their invasive and/or metastatic properties in general [4-12].

Tumour metastasis is a multistep process requiring detachment of malignant cells from the primary tumour, invasion of blood or lymph vessels, interaction with endothelium, extravasation at distant sites and formation of new tumour foci [9,12,13]. It is generally accepted that every step of the metastatic cascade is dependent on specific adhesive interactions of cancer cells with other cells and components of the extracellular matrix. These interactions are mediated by different families of adhesion molecules including cadherins, integrins, members of the immunoglobulin superfamily, and selectins and their carbohydrate ligands – Sialyl Lewis a (sLe^a^) and sLe^x ^[9,13,14].

sLe^x ^is a tetrasaccharide (NeuAcα2 → 3Galβ1 → 4[Fucα1 → 3]GlcNAcβ1 → R) that is particularly relevant from a biological standpoint. It is involved in selectin-mediated adhesion of cancer cells to vascular endothelium and this determinant is thought to be closely associated with hematogenous metastases of cancer [12-17]

In humans, the expression of sLe^x ^is significantly increased in carcinoma cells [4,7,18]. Many clinical studies have shown an association between the expression of sLe^x ^on tumours and enhanced tumour progression and metastasis [7,19]. In woman breast carcinoma the presence of sLe^x ^was also correlated with poor prognosis [20,21]. In fact, the presence of sLe^x ^has been used as a prognostic tumour marker in various types of human cancer [7,19], e.g. lung [22], bladder [8], breast [20,21,23], prostate [24], colon [25] and gastric [26-28] carcinoma. Little is known about the expression of sLe^x ^in canine tumours. To the best of our knowledge only the study of Nakagawa et al describe the expression of sLe^x ^in canine and feline mammary gland tumours [29], but no significant correlation between the expression of sLe^x ^and prognosis has been described in canine or feline tumours.

sLe^x ^and E-cadherin are two adhesion molecules that seem to be involved in malignant progression with opposite roles [31]. Alpaugh et al have described a cooperative role between E-cadherin and sLe^x ^in the passive dissemination of tumour emboli and in the genesis of the lymphovascular embolus of woman's Inflammatory Breast Carcinoma [30-32].

Recently, Jeschke et al identified a negative correlation between Sialyl Lewis antigens and E-cadherin expression in woman breast cancer and their lymph node metastases [23]. This combined analysis of tumour antigens involved in adhesion of breast cancer cells has never been described in canine mammary tumours, which constitutes an important comparative model for woman breast cancer.

The purpose of the present study is to analyse, by immunohistochemical staining methods, the expression of the carbohydrate sLe^x ^in canine malignant mammary tumours and to evaluate the relationship between sLe^x ^expression and tumour clinicopathological features. The relation between the expression of the molecules sLe^x ^and E-Cadherin [33] in canine malignant mammary tumours was also investigated.

## Methods

### Tissue specimens

Fifty-three malignant mammary tumours and 102 local and regional lymph nodes were surgically removed from 35 female dogs aged from 3 to 16 (mean, 9.9 years), of various pure or mixed breeds. The specimens were fixed in 10% neutral buffered formalin. After dehydration and paraffin wax embedment, sections of 4 μm were cut from each representative paraffin blocks for staining with haematoxylin and eosin (HE) and for sLe^x ^and E-cadherin immunohistochemistry (IHC).

Lymph nodes were classified according to the presence of cancer cells (positive or negative), using HE and cytokeratin IHC methods [33].

### Histological examination of the tumours

Tumours, including benign proliferative lesions found in the vicinity of the tumours, were classified independently by two observers from HE-stained sections on the basis of the diagnostic criteria of the World Health Organization classification of tumours in domestic animals [34].

The presence of intra-tumoral necrosis was registered for each case and evaluation and classification of the mode of tumour growth was assessed as previously described [33].

### Follow-up data

All animals were clinically evaluated every 3 months (by physical examination, thoracic radiography and abdominal ultrasound) for the presence of distant metastases during a follow-up period of 2 years after surgery. All dogs were followed until death or until the end of the observation period. In all dogs that died with suspected distant metastases, complete necropsies were performed and histological confirmation was obtained.

### Immunohistochemistry

Canine mammary cancer tissues, which had been resected by the curative operation were routinely processed and used for immunostaining of sLe^x ^and E-cadherin.

Immunostaining was performed by the modified avidin-biotin-peroxidase complex (ABC) method [35]. Two distinct monoclonal antibodies (mAbs) were used: the mAb FH6 generated against sLe^x ^epitope [36] and the monoclonal mouse anti-human E-cadherin antibody (clone 4A2C7, Zymed, S.Francisco, California, USA) [33].

Tumour sections (4 um thick) were deparaffinized in xylene, dehydrated through graded concentrations of ethanol and washed with distilled water. Sections were then treated with Citrate buffer (sodium citrate antigen retrieval solution; 10 mM citric acid, pH = 6) for 20 minutes in a microwave oven at 600 W. The slides were cooled for 10 minutes at room temperature and rinsed twice in Phosphate buffered saline (PBS) for 5 minutes. Endogenous peroxidase activity was blocked by treating the section with hydrogen peroxide 3% in methanol for 10 min. After washing the slides in PBS, non-specific staining was eliminated by incubating the sections with normal rabbit serum (Dako) diluted at 1:5 in PBS containing bovine serum albumin (BSA) 10%, in a humid chamber for 20 min at room temperature. Excess normal serum was removed and replaced by the anti-sLe^x ^mAb FH6 diluted at 1:5. After overnight incubation (≅18 hours) at 4°C, slides were washed with PBS and incubated for 30 min with a 1:200 dilution of biotin-labelled rabbit anti-mouse secondary antibody (Dako). Sections were then washed with PBS and incubated for 30 min with avidin-biotin complex (Dako) diluted at 1:100. This was followed by staining the sections for 5 to 7 minutes with 0.05% 3,3 diaminobenzidinetetrahydrochloride (DAB) freshly prepared in 0.05 M Tris/hydroxymethylaminomethane buffer, pH 7.6, containing 0.1% hydrogen peroxide. Finally, sections were lightly counterstained with haematoxylin, dehydrated, and mounted.

Dilution of primary antibody, biotin-labelled secondary antibody, and avidin-biotin complex were made with PBS containing 5% BSA.

All series included, as positive control sections of a human mixed gastric carcinoma previously shown to display prominent expression of sLe^x^.

Negative controls were performed by substitution of the primary antibody with immunoglobulins of the same subclass and concentration as the monoclonal antibody.

### Scoring of immunostaining and statistical analysis

The degree of mAb FH6 reactivity with individual tissue sections was scored by percentage of stained carcinoma cells in the section by three authors (S.S.P., C.A.R., F.G.) without knowing patients outcome and clinicopathological features of the case. In the event of disagreement, slides were reviewed by the observers, and a consensus was obtained.

Expression of sLe^x ^and E-cadherin [33] in canine mammary carcinomas were classified in the following manner: negative, no immunoreactivity or immunostaining in very rare cells; less than 25% of cancer cells stained; 25–50%, well defined areas with positive cells; 50–75% of cancer cells stained; and more than 75% stained cells. Scoring of immunoreactivity was evaluated irrespectively of localization of positive cells and intensity of the staining.

For statistical analysis the expression of sLe^x ^was regrouped in two percentual categories (<25% and ≥25%) in order to increase the number of cases in each category and in this manner improve the statistical power of the tests.

The statistical relationship among variables was analysed using tables of frequencies and their significance tested by the Fisher's exact test [37]. P values less than 0.05 were considered a significant association.

### Double-labelling Immunofluorescence

For simultaneous visualization of sLe^x ^and E-cadherin on the same tissue section, double-label immunofluorescence was performed. We chose the following representative tissue sections: sections with <25% sLe^x ^and >75% E-Cadherin expression; sections with >75% sLe^x ^and <25% E-Cadherin expression and sections with the same percentage of positive cells for both mAbs.

Paraffin sections were dewaxed, rehydrated and then treated with Extran (Merck, Frankfurt, Germany) 0.05% in distilled water for 10 min in a microwave oven at 750 W. After cooled for 20 min at room temperature, slides were rinsed twice in Phosphate-buffered saline (PBS) and then incubated for 20 min in a humid chamber with rabbit non-immune serum at a dilution 1:5 in PBS containing bovine serum albumin (BSA) 10%. Sections were incubated with the first primary mAb, anti-human E-Cadherin (clone 36, BD Biosciences Pharmigen, diluted 1:100 in PBS), overnight at 4°C. After washing twice for 5 min in PBS, sections were incubated with FITC-conjugated rabbit anti-mouse immunoglobulin (code F261; Dako, Glostrup, Denmark; diluted 1:100 in PBS). Sections were washed two times for 5 min in PBS and blocked with non-immune goat serum diluted 1:5 in PBS containing BSA 10%. Sections were then incubated with mAb FH6 (mouse IgM) diluted at 1:5, overnight at 4°C. Sections were washed two times for 5 min with PBS and incubated with Texas red-conjugated goat anti-mouse IgM (Jackson Immunoresearch) diluted 1:50 in PBS. Sections were washed as before and nuclei were counterstained with 4'-6-Diamidino-2-phenylindole (DAPI, Sigma) for 15 min in the dark, diluted at 1:100 in PBS. Sections were washed two times for 5 min in PBS and mounted with Vectashield (Vector Laboratories, Burlingame, USA).

Dilutions of primary antibodies, secondary antibodies, and DAPI were made with PBS containing 5% BSA.

### Microscopy and image processing

Immunostained slides were examined under a fluorescence microscope (Leica DMIRE2) equipped with appropriate filters. Separate images for DAPI, Texas-Red and FITC staining were captured digitally at ×200 and ×400 magnification. The red (for Texas-Red), blue (for DAPI), and green (for FITC) components were merged and composite images were imported into Adobe Photoshop 7.0^®^.

## Results

### Clinical and pathological features of mammary carcinoma patients

All dogs that were included in this study were female. The mean age at surgery was 9.9 years old (range: 3–16 years old). Histological diagnosis of canine mammary carcinomas consisted of 2 *in situ *carcinomas, 13 complex carcinomas, 13 tubulopappilary carcinomas, 9 solid carcinomas, 1 spindle cell carcinoma, 3 mucinous carcinomas, 10 carcinosarcomas and 2 carcinomas in benign tumour.

The mode of tumour growth was assessed in all of 53 tumours. Invasive tumours were more prevalent (28 cases, 52.8%) followed by expansive tumours (17 cases, 32.1%) and tumours with vessel invasion (8 cases, 15.1%).

Lymph node metastases were evaluated in 50 tumours (in 3 cases the regional lymph nodes were not submitted). The majority of the cases did not show lymph node metastases (39, 78.0%) whereas eleven patients (22.0%) revealed metastases in regional lymph nodes. The majority of the cases that showed lymph node metastases were classified as carcinosarcomas (6 out of 11). The others were classified as complex carcinoma, tubulopapillary carcinoma and solid carcinoma. From the eleven cases that revealed nodal metastases, 4 patients revealed in addition, distant metastases. Seven patients with lymph node metastases died (in 4 animals was performed euthanasia because of the tumour and 3 animals died of other causes).

The presence of intra-tumoral necrosis was observed in thirty tumours (56.6%), whereas 23 (43.4%) tumours did not show this feature.

Of the 35 dogs that were included in this study, 33 completed the follow-up period and ten dogs (30.3%) developed distant metastases. From the 17 dogs that died at the end of the follow-up period, 52,9% died because of the mammary carcinoma and 47,0% died from other causes.

### Expression of Sialyl Lewis x in canine mammary carcinomas

**Figure 1:**
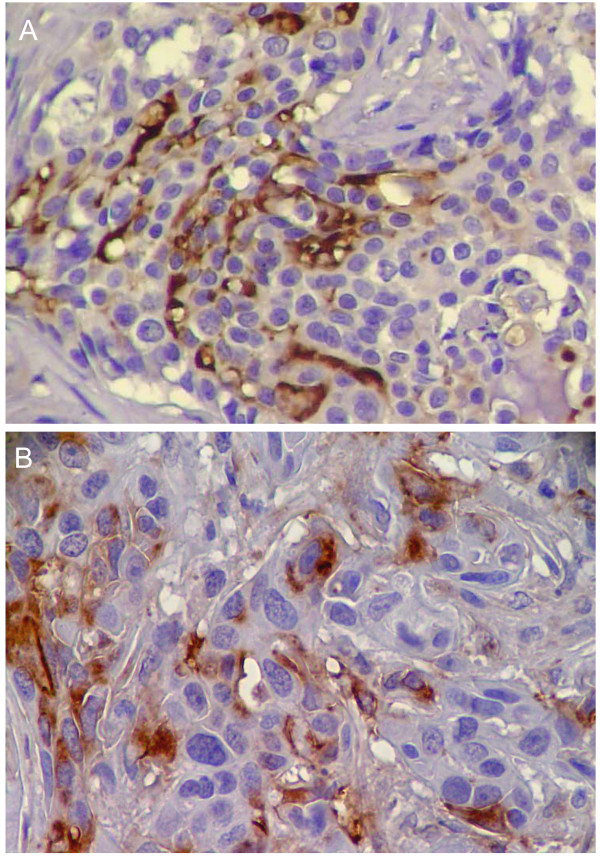
**Immunohistochemical study of the expression of Sialyl Lewis x in canine malignant mammary tumours**. A. Solid carcinoma; >75% of sLe^x ^expression; ×400 B. Carcinosarcoma; 25–50% of cells stained; ×400.

**Figure 2:**
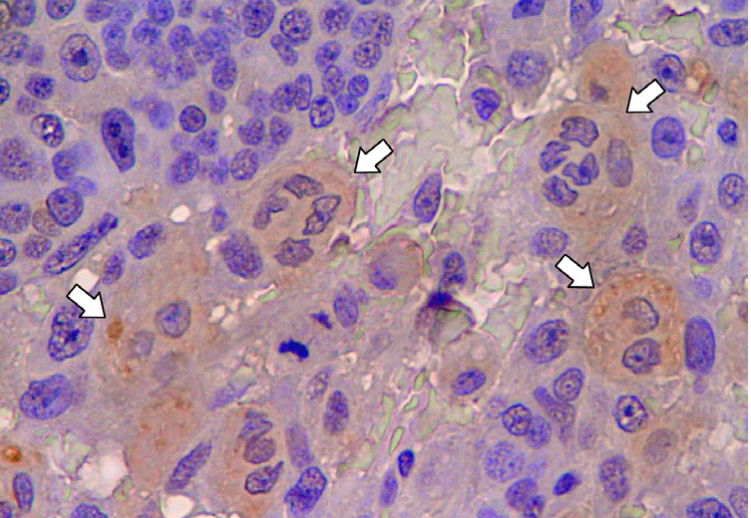
**Immunohistochemical expression of Sialyl Lewis x in squamous metaplasia**. All cells that exhibit squamous metaplasia (arrows) are positive for Sialyl Lewis x. ×400

All of the carcinomas studied by immunohistochemistry showed expression of sLe^x ^antigen (Figure 1). Thirty two (60.4%) were found to express sLe^x ^in less than 25% of the cells, whereas 21 (39.6%) showed sLe^x ^expression in more than 25% of the cells. This distribution of sLe^x ^expression include the early stage cases of canine mammary carcinomas such as *in situ *carcinoma and carcinoma in benign tumour (Table 1). The staining was observed in the cytoplasm and/or in the cell membrane of epithelial cells. In tumour areas with cellular squamous differentiation, sLe^x ^was always expressed (Figure 2). In all cases, the adjacent normal mammary gland tissue and the adjacent benign proliferative lesions were evaluated and they did not showed expression of sLe^x^. It was analysed a total of 43 adjacent benign proliferative lesions (6 simple adenomas, 8 epithelioses and the others were hyperplasias in general). In proliferative lesions sLe^x ^was expressed only in mammary gland secretion.

**Table 1:** Relationship between Sialyl Lewis x expression and clinicopathological features in canine malignant mammary tumours.

		**Sialyl Lewis x expression**		
			
***Clinical Features***	**Number of cases(%)**	**<25%**	**≥25%**	***P *value**
**Histological type **(n = 53)				*NS(0,075)*
In situ carcinoma	2 (3.8%)	1 (50.0%)	1 (50.0%)	
Complex carcinoma	13 (24.5%)	12 (92.3%)	1 (7.7%)	
Tubulopapillary carcinoma	13 (24.5%)	7 (53.8%)	6 (46.2%)	
Solid carcinoma	9 (17.0%)	5 (55.6%)	4 (44.4%)	
Spindle cell carcinoma	1 (1.9%)	1 (100.0%)	0 (0.0%)	
Mucinous carcinoma	3 (5.7%)	1 (33.3%)	2 (66.7%)	
Carcinosarcoma	10 (18.9%)	4 (40.0%)	6 (60.0%)	
Carcinoma in benign tumour	2 (3.8%)	1 (50.0%)	1 (50.0%)	
				
**Mode of Growth **(n = 53)				*NS(0,27)*
Expansive	17 (32.1%)	13 (76.5%)	4 (23.5%)	
Invasive	28 (52.8%)	15 (53.6%)	13 (46.4%)	
Vessel invasion	8 (15.1%)	4 (50.0%)	4 (50.0%)	
				
**Lymph node metastases **(n = 50)*				*0.034*
No	39 (78.0%)	29 (74.4%)	10 (25.6%)	
Yes	11 (22.0%)	4 (36.4%)	7 (63.6%)	
				
**Necrosis **(n = 53)				*NS(0,078)*
Absent	23 (43.4%)	17 (73.9%)	6 (26.1%)	
Present	30 (56.6%)	15 (50.0%)	15 (50.0%)	
				
**Distant Metastasis **(n = 33)				*NS(0,24)*
No	23 (69.7%)	17 (73.9%)	6 (26.1%)	
Yes	10 (30.3%)	5 (50.0%)	5 (50.0%)	

* No lymph nodes were submitted with 3 tumours

NS – Not significant (P > 0.05)

### Relationship between Sialyl Lewis x expression and clinicopathological features

Table 1 summarizes the expression of sLe^x ^according to clinicopathological features.

No significant relationship was found between the expression of sLe^x ^antigen and the different histological types of canine mammary carcinoma according to the World Health Organization classification of tumours in domestic animals [34].

Similarly, no significant relationship was observed between sLe^x ^expression and the growth pattern of mammary carcinomas.

A total of 63.6% of tumours with lymph node metastases showed significantly higher sLe^x ^expression (≥25%), whereas most (74.4%) of the tumours without lymph node metastasis showed underexpression (<25%) of sLe^x ^(p = 0.034).

Although this association did not reach a statistical significance, we observed that tumours without necrosis showed less sLe^x ^expression than tumours with necrosis (p = 0.07).

No significant relationship was found between the expression of sLe^x ^antigen and distant metastases.

### Relationship between Sialyl Lewis x and E-cadherin expression

**Figure 3:**
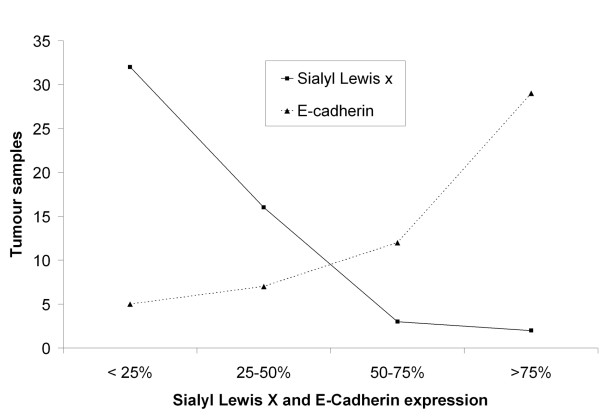
**Relation between Sialyl Lewis x and E-cadherin expression. **The figure illustrates the negative correlation between sLe^x ^and E-Cadherin expression in canine malignant mammary tumours. When expression of E-cadherin increase, the expression of sLe^x ^decrease and vice-versa.

The analysis of expression of sLe^x ^and E-cadherin disclosed an inverse correlation among the two molecules, higher sLe^x ^expression was accompanied with lower E-cadherin expression and vice-versa (Figure 3). When mammary carcinomas show underexpression of E-cadherin (<25%), all of them simultaneously revealed a higher sLe^x ^expression (≥25%). On the other hand when tumour samples showed overexpression of E-cadherin (>75%) the majority of them (93.1%) simultaneously revealed less than 50% of sLe^x ^expression. In summary, we found a significant relationship (p = 0.013) between sLe^x ^and E-cadherin expression in canine malignant mammary tumours (Table 2).

**Table 2:** Relationship between Sialyl Lewis x and E-cadherin expression in canine malignant mammary tumours.

		**Sialyl Lewis x expression**		
			
**E-Cadherin expression**	**Number of cases (%)**	**<25%**	**≥25%**	***P *value**
<25%	5 (9.4%)	0 (0.0%)	5 (100.0%)	0.013
25–50%	7 (13.2%)	6 (85.7%)	1 (14.3%)	
50–75%	12 (22.6%)	9 (75.0%)	3 (25.0%)	
>75%	29 (54.7%)	17 (58.6%)	12 (41.4%)	

**Figure 4:**
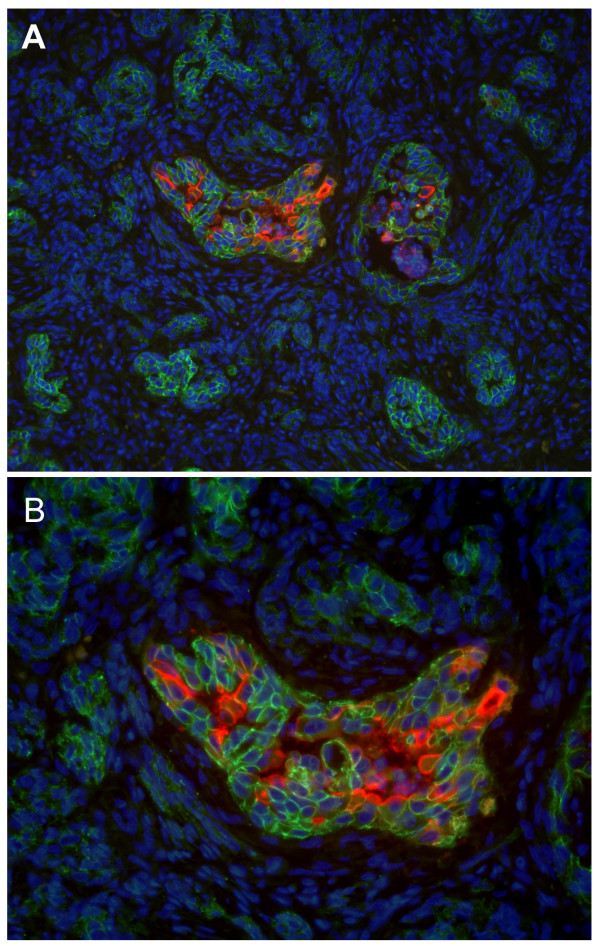
**Double-label immunofluorescence of canine malignant mammary tumour (Complex carcinoma)**. A – Cells that are positive for Sialyl Lewis x (red) are negative for E-cadherin (green) and vice-versa. ×200 B – Absence of co-expression of sLe^x ^and E-cadherin in the same cells. ×400

Simultaneous expression of sLe^x ^and E-cadherin was analysed using double-label immunofluorescence method (Figure 4), demonstrating the absence of overlapping between the two molecules (Figure 4). Based on these results, it seems that, when cells express sLe^x ^they do not express E-cadherin, and on the other hand when they are positive for E-cadherin they are negative for sLe^x^.

## Discussion

Malignant transformation of tumour cells is associated with abnormal glycosylation, resulting in expression of altered carbohydrate determinants including the expression of sLe^x ^and sLe^a ^antigens [4,9,12]. These carbohydrate antigens have been shown to be useful tumour markers in carcinomas of different organs [4,9,12]. The biosynthesis of these oligosaccharides are usually increased and altered during the acquisition of the malignant phenotype and tumour progression [5,12]. Some studies refer that aberrant glycosylation is a result of initial oncogenic transformation, as well as a key event in induction of invasion and metastases [10,12].

Most human carcinomas show changes within cell surface carbohydrates compared to their normal counterparts [4,12].

sLe^x^, present on the surface of tumour cells has been found to serve as ligands for endothelial E-selectin and was shown to play a major role in the process of adhesion of cancer cells to the endothelium [38]. sLe^x ^not only is a marker for cancer but also is functionally implicated in the malignant behaviour of cancer cells [4,12-14]. E-selectin, one of the selectin family member is expressed on the vascular endothelial cells and adheres to a carbohydrate ligand, sLe^x ^[15,16]. Binding of sLe^x^, present on the cell membrane of neutrophil granulocytes, to E-selectin, expressed on activated endothelial cells, was shown to initiate neutrophil extravasation and migration into tissues [15]. It has been hypothesized that the E-selectin/sLe^x ^interaction can mediate the sequence of adhesion of tumour cells to endothelium and their subsequent extravasation in a process that mimics the above process of inflammation [15,17]. The sLe^x ^antigen is expressed by various human carcinomas such as: gastric carcinoma [26], colorectal carcinoma [25], lung cancer [22], prostate carcinoma [24], bladder carcinoma [8], oral [39], head&neck squamous cell carcinoma [40] and breast cancer [11,20,23]. The amounts of sLe^x ^expression are closely associated with progression and with poor prognosis in those cancers.

In canine cancers, Nakagawa et al has described the expression of sLe^x ^in canine and feline mammary gland tumours. However no association with clinicopathological features and prognosis was observed [29,41].

The present study demonstrates that in malignant canine mammary tumours the malignant transformation of the mammary gland is accompanied by expression of sLe^x^. These results are in agreement with previous observations showing that in squamous metaplasia, used as criteria of malignancy in canine mammary cancer [42-44], there is a strong expression of sLe^x^antigen. These observations support the use of sLe^x ^as a prognostic tumour marker in canine mammary carcinomas.

In this study, we found no significant relationship between sLe^x ^expression and the histological type of the tumours, classified according to World Health Organization classification. This result is in agreement with the results described by Nakagawa et al in canine and feline mammary gland tumours [29]. Similarly, in woman breast cancer, Nakagoe et al did not find an association between sLe^x ^expression and histological type of the carcinoma [20].

Previous studies have shown that, in woman breast cancer, tumour necrosis is associated with poorer survival [45-47] and higher recurrence rates [46]. In canine mammary tumours, the presence of large areas of necrosis within the tumour mass could be used as criteria for a diagnosis of malignancy [44]. In spite of no association between sLe^x ^expression and tumour necrosis we observed that some tumours with necrosis showed high amounts of sLe^x ^expression.

It is clear that the expression of sLe^x ^in tumours is a marker of the invasive and/or metastatic properties of the tumours [4,11]. In addition, it has been shown that sLe^x ^promotes binding of tumour cells at an invasion focus to endothelial cells through E-Selectin [11]. Many clinical studies show a clear association between the expression of sLe^x ^and tumours with enhanced progression and metastases [19]. In woman breast cancer, it was reported that the expression of sLe^x ^antigen in tumour cells was associated with poorer prognosis [20]. Matsuura et al demonstrated that the level of sLe^x ^was elevated in the sera of patients with metastatic breast cancers [21]. Still in woman breast cancer, Jeschke et al reports that overexpression of sLe^x ^was associated with poorer prognosis and malignant relapse [23].

To date, the expression of sLe^x ^in canine malignant mammary tumours, was not correlated neither with clinicopathological features, neither with prognoses [29].

In the present study we found a significant correlation between sLe^x ^expression and lymph node metastases. To the best of our knowledge, this is the first study to show such a relationship in canine malignant mammary tumours. These observations may suggest that sLe^x ^expression may also play a role in the process of local lymphatic invasion and metastization [38]. On the other hand, we did not observe an association between sLe^x ^expression and the development of distant metastases. This observation may suggest that in canine mammary tumours sLe^x ^does not contribute to haematogenous metastasis. However we could not rule out that the absence of association of sLe^x ^and haematogenous metastasis stems from the small number of cases studied for this clinicopathological feature.

These combined results from the relation between sLe^x ^expression and the clinicopathological features allow us to conclude that the sLe^x ^carbohydrate structure contributes for the malignant phenotype and tumour progression of canine malignant mammary tumours.

Tumour cell dissemination and development of metastases is a multistep process involving complex interaction between cancer cells, extracellular matrix, the vascular system, the immune system and the target organs [48]. Adhesion molecules are contributory factors toward metastatic activity. Adhesion can be divided into reduced adhesion of tumour cell, tumour cell interaction and increased adhesion of floating tumour cells to vascular endothelial cells [48]. Recently we and other authors have described that the loss of E-Cadherin expression may have prognostic value in canine malignant mammary tumours [33,49]. In the present study, our results also showed that increasing expression of sLe^x ^correlates with lymph node metastases in canine mammary carcinomas. To address a possible cooperative role between these two molecules involved in cell adhesion, sLe^x ^and E-cadherin, we have compared their expression in canine malignant mammary tumours. Our results showed an inverse relationship between sLe^x ^and E-cadherin expression. Cases expressing sLe^x ^showed decrease E-Cadherin expression and vice-versa. Similarly, we could observe in doubled labelled immunofluorescence images, that cells that expressed sLe^x ^were negative for E-cadherin and vice-versa. This observation is the first indication of a significant relationship between sLe^x ^and E-cadherin expression in canine malignant mammary tumours. Based on our results it seems that the expression of sLe^x ^and/or E-cadherin on the same tumour cell could be regulated by an internal mechanism of the cell. In a previous study in a model of woman Inflammatory Breast Carcinoma (IBC), Alpaugh et al have described a cooperative role of E-cadherin overexpression and sLe^x ^underexpression in the genesis of the lymphovascular embolus of IBC [30,31]. These results showed that both types of adhesion molecules might play opposing roles within the same tumour cell model. Furthermore, Jeschke et al have also shown, in breast cancer, a negative correlation between the expression of Sialyl Lewis antigens and E-cadherin as the risk of breast cancer metastasis progresses [23].

Our findings, in canine mammary carcinomas, supports the generally accepted dogma that metastatic propensity is an active biologic process that includes the decrease of tumour cells adhesion at the primary tumour site (E-cadherin down-regulation) which is thought to be accompanied by higher mobility and invasiveness [50] contributing to the process of local lymphatic invasion and metastization (sLe^x ^overexpression).

## Conclusion

Our results demonstrate the importance of sLe^x ^in malignant phenotype of canine mammary carcinoma and support the use of sLe^x ^as a prognostic tumour marker in canine malignant mammary gland tumours.

The significant relation between sLe^x ^and lymph node metastases allow us to conclude that sLe^x ^can be used as prognostic factor in canine malignant mammary tumours.

There is an inverse relationship between the expression of the adhesion molecules sLe^x ^and E-cadherin, in canine mammary carcinomas.

The present results warrants further investigation addressing the clarification of the molecular mechanism determining this inverse correlation between sLe^x ^and E-cadherin as well as the investigation of the mechanisms by which sLe^x ^interferes with homotypic cellular adhesion mediated by E-cadherin in canine malignant mammary tumours, as a comparative model to woman breast cancer.

## Abbreviations

sLe^x ^= Sialyl Lewis x; sLe^a ^= Sialyl Lewis a; HE = Haematoxylin and eosin; IHC = Immunohistochemistry; ABC = Avidin-biotin-peroxidase-complex; mAbs = Monoclonal antibodies; PBS = Phosphate buffered saline; BSA = Bovine serum albumin; DAB = 3,3diaminobenzidinetetrahydrochloride; DAPI = 4'-6-Diamidino-2-phenylindole; NS = Non significant; IBC = Inflammatory Breast Carcinoma

## Competing interests

The author(s) declare that they have no competing interests.

## Authors' contributions

SSP and AJFM performed the study. SSP wrote the manuscript. CL participated in the immunohistochemistry method. NTM participated in the composition of the images. SSP and JC carried out the statistical analysis. CAR and FG participated in the design and coordination of the studies and contributed strongly to the revision of the manuscript. All the authors read and approved the final manuscript.

## Pre-publication history

The pre-publication history for this paper can be accessed here:


